# Opposing gastric and jejunal regulation of CELA2A in obesity and after Roux-en-Y gastric bypass suggests a role in gastrointestinal metabolic signaling

**DOI:** 10.3389/fendo.2026.1833946

**Published:** 2026-05-25

**Authors:** Carl I. W. Larson, Melania Aluia, Eric Schéle, Suzanne L. Dickson, Anna Casselbrant, Ville Wallenius

**Affiliations:** 1 Department of Surgery, Institute of Clinical Sciences, Sahlgrenska Academy at the University of Gothenburg, Gothenburg, Sweden; 2 Department of Neuroscience and Physiology, Sahlgrenska Academy at the University of Gothenburg, Gothenburg, Sweden; 3 Region Västra Götaland, Department of Surgery, Sahlgrenska University Hospital Östra, Gothenburg, Sweden

**Keywords:** bariatric surgery, CELA2A, gastrointestinal physiology, metabolic regulation, Roux-en-Y gastric bypass, serine protease, small intestine, glycemic control

## Abstract

**Aims:**

Roux-en-Y gastric bypass (RYGB) rapidly improves glycemic control in obesity and type 2 diabetes (T2D), but the underlying mechanisms remain incompletely understood. Chymotrypsin-like elastase family member 2A (CELA2A), a circulating pancreatic serine protease implicated in metabolic regulation, has been proposed to contribute to these effects, although its regulation in obesity and after metabolic surgery remains unclear. We investigated CELA2A expression in gastrointestinal tissues and circulation in relation to obesity and RYGB.

**Methods:**

Gastric biopsies were collected in a cross-cohort, non-paired comparison perioperatively from patients undergoing sleeve gastrectomy and postoperatively from patients after RYGB. Jejunal biopsies and serum samples were obtained in paired study setup before and after RYGB. Intestinal tissue and serum were also analyzed in mice fed a control or high-fat diet. CELA2A expression was assessed using Western blot, immunohistochemistry, and ELISA.

**Results:**

After RYGB, CELA2A expression increased in gastric mucosa but decreased in the jejunum, accompanied by reduced circulating CELA2A. Before surgery, jejunal CELA2A levels were positively associated with HbA1c, but not with fasting insulin. In high-fat diet–fed mice, jejunal CELA2A expression was increased, whereas gastric expression was reduced. Circulating CELA2A levels were not significantly altered in this model. In both species, CELA2A immunoreactivity was observed in mucosal glandular structures of the stomach and jejunum; however, detailed cellular localization in human tissue was not established, and circulating and tissue levels were not directly correlated.

**Conclusion:**

CELA2A exhibits tissue-specific regulation in obesity and following bariatric surgery and may represent a candidate component of gastrointestinal metabolic signaling. Further studies are required to define its cellular origin and functional role in glucose homeostasis.

## Introduction

Obesity represents one of the most pressing global health challenges. Its prevalence continues to rise worldwide and is strongly associated with type 2 diabetes (T2D), cardiovascular disease, and increased mortality (1). Individuals with severe obesity (BMI ≥35 kg/m²) are at particularly high risk of metabolic and cardiovascular complications, underscoring the need for effective and durable therapeutic strategies (2).

Bariatric surgery is the most effective treatment for severe obesity, leading not only to sustained weight loss but also to profound metabolic improvement (3–5). Notably, procedures such as Roux-en-Y gastric bypass (RYGB) induce rapid remission of type 2 diabetes (T2D), often within days to weeks after surgery and well before significant weight loss occurs (6–10). This temporal dissociation indicates that the antidiabetic effects of RYGB are not solely attributable to reduced adiposity, but rather reflect immediate, surgery-induced alterations in nutrient handling and gut-derived signaling pathways.

Several mechanisms have been proposed to explain these early effects, including enhanced secretion of incretin hormones, altered bile acid metabolism, remodeling of the intestinal microbiota, and rapid improvements in insulin sensitivity (11–14). In particular, RYGB markedly enhances postprandial secretion of glucagon-like peptide-1 (GLP-1), contributing to improved insulin secretion and glycemic control (6, 15). The foregut and hindgut hypotheses further propose that exclusion of the proximal small intestine or accelerated nutrient delivery to the distal intestine drives these metabolic improvements through altered enteroendocrine signaling (9, 16). According to the foregut hypothesis, bypass of the stomach, duodenum, or proximal jejunum may suppress the release of a putative diabetogenic or incretin-modulating factor that is normally stimulated by nutrient exposure in these segments (9). Despite extensive investigation, however, the molecular mediators underlying these early, weight-independent metabolic adaptations remain incompletely defined.

Recent proteomic analyses of the gastric mucosa following RYGB identified several proteins exhibiting differential postoperative abundance. Among these was chymotrypsin-like elastase family member 2A (CELA2A), a serine protease traditionally characterized as a pancreatic digestive enzyme (17). Although initially described as a zymogen secreted from the exocrine pancreas (18, 19), CELA2A has more recently been detected in human plasma, suggesting additional endocrine or paracrine functions (20). Emerging evidence links CELA2A to metabolic regulation: loss-of-function mutations have been associated with impaired insulin secretion, reduced insulin sensitivity, platelet activation, early-onset atherosclerosis and features of the metabolic syndrome (20). Genome-wide association studies have implicated CELA2A among rare variants linked to hypertension and other cardiometabolic traits (21). These findings suggest that CELA2A may participate in systemic metabolic control rather than serving solely as a digestive enzyme.

Despite these associations, the tissue-specific expression patterns and systemic dynamics of CELA2A in obesity and after bariatric surgery remain largely unexplored. In particular, it is unknown whether CELA2A is differentially regulated along the gastrointestinal tract in response to metabolic stress, whether its circulating levels reflect compensatory or pathogenic processes, or whether its modulation contributes to the metabolic remodeling observed after RYGB. The fundus and corpus of the stomach are very similar histologically and functionally, but there are subtle differences. The main effect of the fundus has been considered storage and regulation of intragastric pressure but is less active in mixing and propulsion, and the fundus is the major site for ghrelin production. The corpus´s main role has been considered acid and enzyme secretion and mixing of food and gastric juices. We now wanted to examine if there were any regional differences regarding CELA2A production.

The primary aim of the present study was therefore to characterize CELA2A expression as a potential “foregut factor” in the stomach, jejunum, and circulation in relation to obesity and RYGB. We examined gastric and jejunal biopsies and serum samples from bariatric surgery patients before and after RYGB in two different cohorts, gastric biopsies in a cross-cohort, non-paired comparison, and jejunal biopsies and serum samples before preoperative diet, at surgery and postoperatively by enteroscopy in a paired study setup. To complement these findings and explore the influence of diet-induced metabolic stress, we further assessed CELA2A expression in gastrointestinal tissues and circulation of control and high-fat diet–fed mice. By integrating human and experimental data, we sought to determine whether CELA2A exhibits tissue-specific and context-dependent regulation in obesity and after bariatric surgery.

## Materials and methods

### Human subjects and ethics

Patients planned for sleeve gastrectomy, RYGB or postoperatively after RYGB for enteroscopy willing to participate gave written informed consent and were enrolled in the study at Sahlgrenska University Hospital in Gothenburg, Sweden. A total of 34 patients agreed to participate in the two experimental setups. Overall patient information is shown in **Table 1**. The study was approved by the Regional Ethical Review Board in Gothenburg, Sweden (Dnr. 2019−00337 and Dnr. 007–09) and conducted in accordance with the Declaration of Helsinki.

**Table 1:** Patients.

Baseline and postoperative characteristics of the study cohorts	Baseline	Perioperative/After LED	Post surgery
Sleeve Gastrectomy (Obese periop gastric biopsies)			
N		6	
Gender (f/m)		4/2	
Age (years)		41 [29-54]	
BMI (kg/m²)		44 ± 2.7	
Number of T2D		1	
Post-RYGB (postop gastric biopsies)			
N			6
Gender (f/m)			2/4
Age (years)			53 [50-59]
BMI (kg/m²)			27 ± 2.1
Number of T2D			0
Pre+post-RYGB (jejunal biopsies)			
N	22	12	12
Gender (f/m)	13/9	8/4	8/4
Age (years)	52 [34-69]	52	52
BMI (kg/m²)	40 ± 0.4	37 ± 0.7	30 ± 0.7
Number of T2D	12	5	2

BMI, Body Mass Index; T2D, Type 2 Diabetes Mellitus; RYGB, Roux-en-Y Gastric Bypass; LED, Low-Energy Diet. Mean ± SEM or range.

Baseline gastric mucosa biopsies were retrieved from the fundus, corpus and antrum perioperatively from the excised part of the stomach from a total of six patients undergoing sleeve gastrectomy. The inclusion criteria were BMI ≥ 35 kg/m2 with or without T2DM and/or hypertension. Exclusion criteria were not being able to understand the Swedish language or if the mucosa showed manifestations of any macroscopic signs of disease, e. g. inflammation, polyps or ulcers.

For postoperative biopsies, single-balloon enteroscopy was performed in intubated and anesthetized RYGB patients that were planned for enteroscopy for clinical diagnostic purposes, mainly chronic abdominal pain where no clear cause had been found by usual clinical examinations (e.g. gastroscopy, biliary ultrasound, abdominal CT scan, barium swallow, diagnostic laparoscopy). The biopsies from the excluded stomach after RYGB were obtained using a single-balloon enteroscope (Olympus SIF-Q180, Olympus Medical Systems, Tokyo, Japan) which was introduced via the esophagus to the gastric remnant and through the gastroenterostomy to the Roux limb and jejunojejunostomy, and then in a retrograde fashion via the duodenum and pylorus enabling access to the gastric remnant. We have recently published pictures taken from the gastric remnant though the enteroscope (17). The route for the ballon-enteroscopy to reach the secluded stomach is also depicted in the Graphical abstract (**Supplementary Figure 1**). The findings in the subjects chosen for this study were all judged normal on the balloon-enteroscopy examination devoid of any clear intestinal pathology.

After reaching the secluded stomach with the single-balloon enteroscope, standard endoscopic biopsy forceps were used to obtain the tissue samples from the fundus, corpus, and antrum. Thus, the biopsies were collected in a non-paired fashion to the baseline perioperative sleeve gastrectomy samples. This approach was necessitated by ethical and practical considerations, since it was not possible to perform the single-balloon enteroscopies in anesthetized patients solely for research purposes. Consequently, the interval between the RYGB operation and the endoscopic examination and the biopsies ranged from 8 months to 8 years. A total of six patients were included in this group of postoperative samples.

The preoperative jejunal biopsies were obtained endoscopically from 22 subjects before the 2-3-week long low-energy diet (LED) treatment preceding RYGB (**Table 1**). Perioperative jejunal samples were collected in a subset of 12 of the same individuals during surgery 50–60 cm distal to the ligament of Treitz when dividing the omega-loop during laparoscopic construction of the RYGB. Finally, postoperative biopsies from these 12 individuals were taken 6–8 months after surgery by endoscopy from the alimentary limb close to the portion that was stapled off perioperatively during surgery. Simultaneously with the biopsies venous blood samples were collected at each occasion and f-glucose, f-insulin and HbA1c were measured at the central hospital laboratory at the Sahlgrenska University Hospital. Of samples retrieved from the 12 patients, six had T2D before surgery (HbA1c ≥ 45 mmol/mol) and six were nondiabetic. These were considered an exploratory subgroup. Three to five mucosal biopsies from each level were snap frozen in liquid nitrogen or chemically fixed for later analysis during all these samplings.

### Experimental animals and ethics

Male and female C57BL/6J mice (5–6 weeks old) were obtained from Charles River (Germany). Animals were housed at the Experimental Biomedicine (EBM) facility of the University of Gothenburg under standard conditions (12 h light/dark cycle, temperature- and humidity-controlled environment) with ad libitum access to food and water. All animal experiments were approved by the Regional Animal Ethics Committee in Gothenburg, Sweden (Dnr. 5.8.18-02501/2025) and were performed in accordance with the European Directive 2010/63/EU on the protection of animals used for scientific purposes.

### Feeding intervention

Five- to six-week old mice were randomly assigned to one of two groups: a control group, maintained on a standard chow diet (normal chow, NC; 3.1 kcal/g per 100 g of product), or a high-fat diet group (HFD; 60% kcal from fat, 25% kcal from sugar, 15% kcal from processed food; 5.24 kcal/g per 100 g of product; Diet Lot 25021006, Research Diets, Inc., USA) for 4 weeks. Body weight, food intake, and blood glucose levels were monitored weekly. When the mice in the HFD group were euthanized after 4 weeks they exhibited an average 24% increase in body weight compared to their initial weight, whereas the NC mice only increased an average 11% in body weight. Mice were euthanized using an isoflurane overdose. Blood was collected for plasma and serum isolation, and tissues including small intestine and stomach were harvested for subsequent analyses.

### Proteomics analysis on human gastric mucosa

Previous proteomic analyses of the gastric mucosa from the fundus, corpus, and antrum were conducted in our laboratory using PRIDE dataset (http://www.ebi.ac.uk/pride) under accession number (PXD065508) (22).

### Western blot

Stomach (antrum, corpus, and fundus) and jejunum tissues from both human and mouse samples were homogenized by sonication, while plasma and serum samples were diluted in ice-cold protein extraction buffer (10 mM potassium phosphate buffer, pH 6.8, containing 1 mM EDTA and 10 mM 3-[(3-cholamidopropyl) dimethylammonio]-1-propanesulfonate [CHAPS]), supplemented with Complete™ protease inhibitor cocktail (Roche, version 12). Protein extracts were separated by SDS–PAGE using 4–15% Criterion™ TGX Stain-Free™ precast gels (Bio-Rad, Solna, Sweden) and subsequently transferred onto low-fluorescence PVDF membranes (Bio-Rad) with a Trans-Blot Turbo transfer system (Bio-Rad). Membranes were blocked with 0.2% (w/v) I-Block™ protein-based blocking reagent (10 mL per membrane; Thermo Fisher Scientific, Uppsala, Sweden) and incubated overnight at 4 °C with a primary antibody against CELA2A (for human samples: #23050S, 1:500, Cell Signaling Technology, and for mouse samples: PA5-116732, 1:500, Thermo Fisher). After washing with block buffer, membranes were incubated with horseradish peroxidase (HRP)-conjugated secondary antibodies (1:2000; Cell Signalling Technology, Danvers, MA, USA). Immunoreactive bands were visualized using enhanced chemiluminescence and imaged with a ChemiDoc™ MP Imaging System (Bio-Rad). Band intensities were quantified by densitometric analysis and normalized using a stain-free total protein normalization method. The primary antibody was selected based on reported specificity for CELA2A; however, given the sequence homology within the chymotrypsin-like elastase family, potential cross-reactivity with related isoforms (e.g., CELA3A/CELA3B) cannot be excluded.

### ELISA

Blood samples collected from patients at the designated time points (see above) were processed to obtain serum, aliquoted, and stored at −80 °C. CELA2A levels in serum samples were quantified using Human CELA2A/ELA2A ELISA techniques (LS-F66699-1, LifeSpan BioSciences, Newark, CA, USA) according to the manufacturer instructions.

### Immunohistochemistry

The chemically fixed samples were prepared and embedded in paraffin. The cut slices were deparaffinized with tissue clear and rehydrated through graded ethanol (99.5% ethanol followed by 95% ethanol). Slides were then washed in 1x PBS and boiled in a citrate buffer (pH 6.0) before being cooled to room temperature and again washed in 1x PBS. A blocking solution (5% goat-serum and 1X PBS + 0.3% Triton X-100; Sigma Aldrich, Saint Louis, MO, USA) was applied and allowed to sit for 2 hours. Primary antibody against CELA2A (PA5-116732, 1:200, Invitrogen, Carlsbad, CA, USA) was then applied to all the biopsies except the negative controls, which remained in blocking solution, and the slides were incubated overnight at 4 °C. The slides were then washed twice in 1X PBS + 0.3% Triton X-100 before each biopsy was incubated with a secondary antibody, Alexa FluorTM 488 goat anti-rabbit (1:500, A11008, Invitrogen), for 2 hours in darkness. After washing, Hoechst 33258 (1:1000, Life Technologies, Carlsbad, CA, USA) was added for 10 min, then washed again before cover glass with ProLongTM Gold Antifade Reagent (Life Technologies) was mounted over the tissue sections. Slides were analyzed using a fluorescence microscopy (Leica Microsystems, Wetzlar, Germany).

### Statistical analysis

Data distribution was assessed using Shapiro–Wilk normality test. Parametric (paired or unpaired *t*-tests) or non-parametric tests (Mann–Whitney or Kruskal–Wallis) were applied accordingly. When applicable, Friedman’s test, Dunn’s multiple comparisons test, and Wilcoxon’s test were used for paired analyses. Outliers were identified using Grubbs’ test (α = 0.05). Simple linear regression was used to analyze the associations between CELA2A expression and f-glucose, f-glucose and HbA1c. Log-transformed Welch’s test was used for analysis of CELA2A jejunal baseline levels in the diabetics vs. non-diabetics. All statistical analyses were performed using GraphPad Prism (v.10.4.2; GraphPad Software Inc.). Differences were considered statistically significant at *p* < 0.05 (*p < 0.05; **p < 0.01). All data are presented as mean ± SEM.

## Results

### RYGB upregulates gastric CELA2A expression in humans

**Figure 1:**
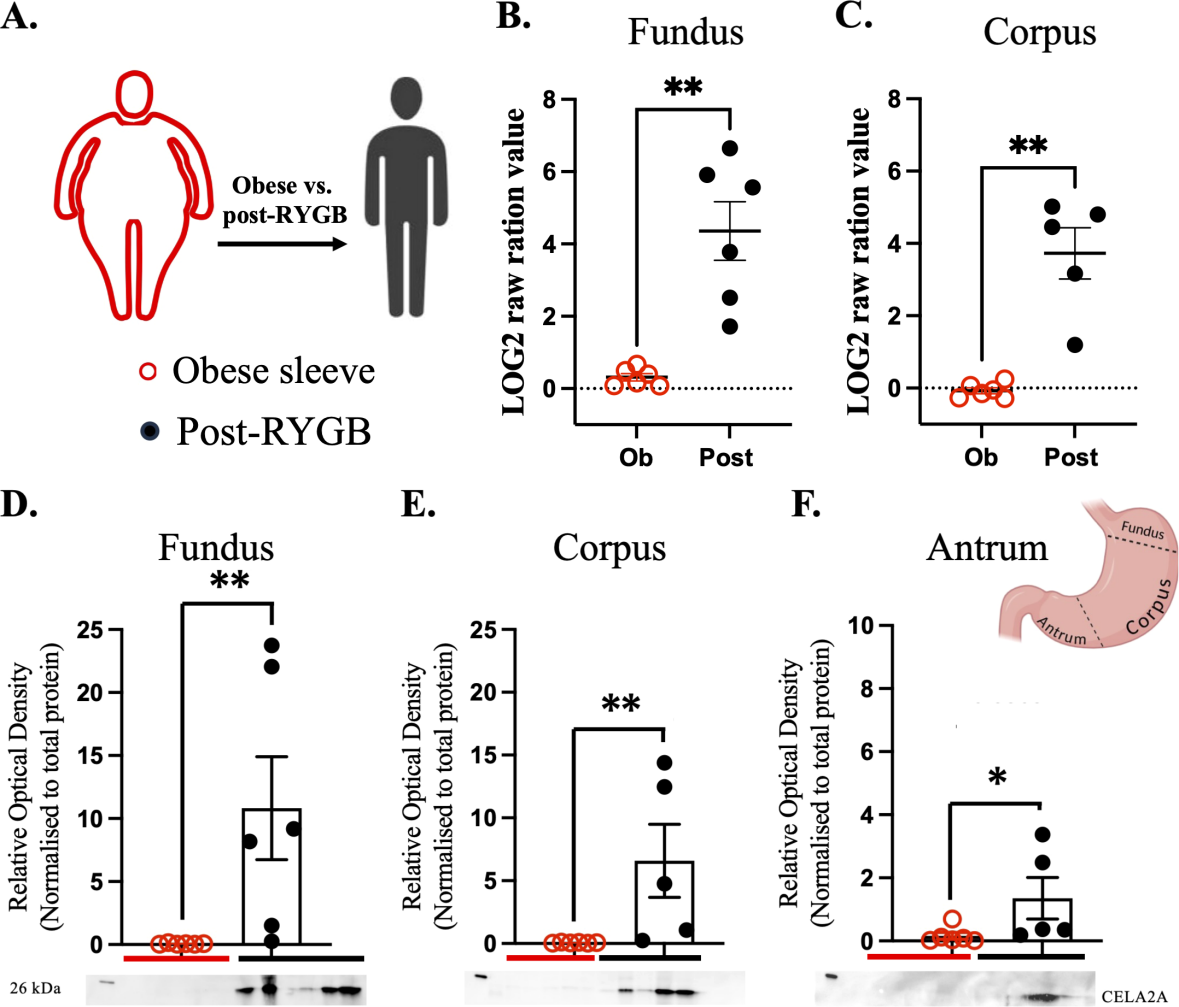
Expression of CELA2A in the human stomach. **(A)** Experimental design: gastric tissue samples were obtained from obese individuals undergoing sleeve gastrectomy, as well as postoperatively from RYGB operated individuals by balloon-enteroscopy. **(B, C)** Individual protein expressions analyzed by proteomic analysis in the fundus **(B)** and corpus **(C)**. **(D-F)** Protein expression assessed by Western blot in the fundus **(D)**, corpus **(E)** and antrum **(F)**. RYGB: Roux-en-Y gastric bypass. N = 6. Significance was calculated using Mann-Whitney U-test. Data are presented as mean ± SEM. *p<0.05 and **p<0.01. llustrations Created with BioRender.com.

In our previous proteomic analysis, we reported that CELA2A expression in the gastric mucosa of the fundus and corpus increased by more than 50% following Roux-en-Y gastric bypass (RYGB) (17). In the present study, we extended these findings by examining individual-level proteomic profiles in subjects with obesity (BMI 44 ± 2.7 kg/m²) before and after RYGB (BMI 27 ± 2.1 kg/m²). CELA2A expression was significantly increased postoperatively in both the fundus (p = 0.0022) and corpus (p = 0.0043) (**Figures 1B, C**). In contrast, proteomic analysis of the antrum did not detect CELA2A but instead identified CELA3A and CELA3B (data not shown).

To validate these observations, CELA2A protein levels were assessed by Western blot across all gastric regions. A distinct band consistent with CELA2A was detected at approximately 26 kDa. Quantitative analysis confirmed significant postoperative upregulation in the fundus (p = 0.0022) and corpus (p = 0.0043), with a more modest but significant increase in the antrum (p = 0.0303) (**Figures 1D–F**). Across regions, CELA2A expression exhibited a decreasing gradient from fundus to corpus to antrum. Since the time after RYGB surgery when biopsies were taken varied between 8 months and 8 years, we also tested whether CELA2A expression was dependent on time elapsed since surgery but found no such correlation (**Supplementary Figure 1**).

### Jejunal and circulating CELA2A levels decrease after RYGB

Given the extensive intestinal remodeling following RYGB, we next examined CELA2A expression in the jejunum in a longitudinal cohort of twelve patients undergoing RYGB, six with type 2 diabetes (T2D) and six without. Jejunal biopsies were obtained at three time points: prior to initiation of a very low-energy diet (LED) (BMI 40 ± 0.6 kg/m², n=12), after completion of the diet at the time of surgery (BMI 37 ± 0.7 kg/m², p < 0.0005 vs. baseline), and 6–8 months postoperatively (BMI 30 ± 0.7 kg/m², p < 0.002 vs. baseline).

**Figure 2:**
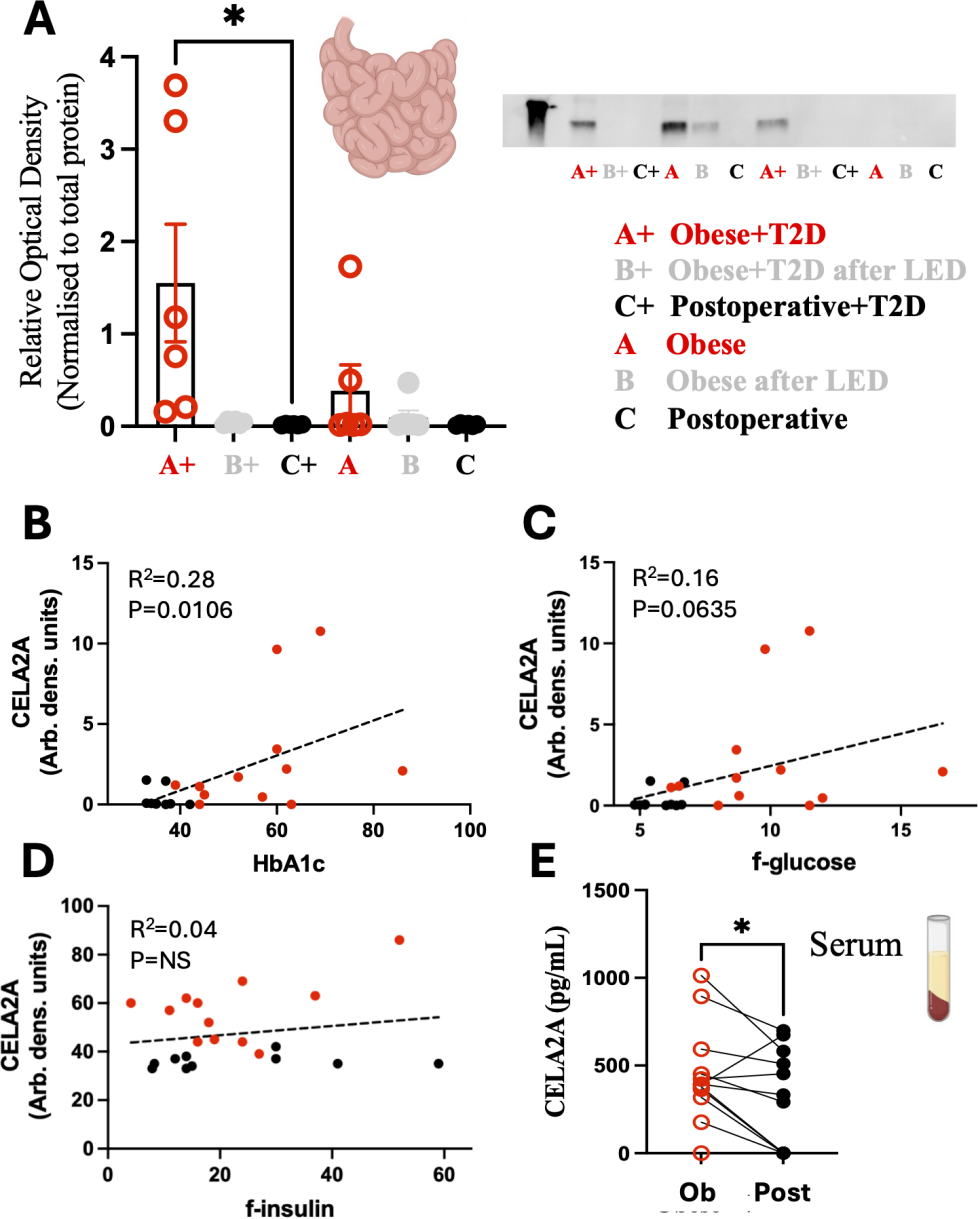
Expression of CELA2A in the human jejunum. **(A)** Jejunal tissue samples were obtained by enteroscopy from obese individuals before preoperative LED-diet **(A)**, as well as 6–8 months postoperatively after RYGB **(C)**. Perioperative samples were retrieved by stapling off a few cm´s of the jejunum when dividing the omega loop during RYGB surgery **(B)**. **(B)** Jejunal CELA2A was significantly associated with HbA1c in the baseline samples before preop LED-diet. **(C)** There was also a tendency to an association between jejunal CELA2A and f-glucose at baseline. **(D)** Jejunal CELA2A and f-insulin showed no association. **(E)** Serum concentration of CELA2A determined by ELISA before and after RYGB. RYGB: Roux-en-Y gastric bypass. T2D, type 2 diabetes. N = 22 in B-D, and 12 in **(A, E)** Significance was calculated using Wilcoxon signed-rank test. Simple linear regression was used in **(B–D)**. Data are presented as mean ± SEM. *p<0.05. Red dots in panels **(B–D)** represent subjects with T2D, black dots represent non-diabetics. llustrations Created with BioRender.com.

Distinct bands consistent with CELA2A were detected in eight of twelve patients. Among these, only two individuals exhibited CELA2A expression in both the pre-LED and perioperative samples. Semiquantitative analysis demonstrated a significant reduction in CELA2A expression after RYGB that was observed primarily among patients with T2D (p = 0.0312), although subgroup size was limited. No postoperative sample from any participant displayed prominent CELA2A bands (**Figure 2A**).

For analysis of possible metabolic associations we had jejunal samples from 10 additional untreated (pre-LED) obese subjects available (BMI 40 ± 0.4 kg/m², n=22). In this expanded cohort of 22 patients, jejunal CELA2A levels were positively associated with HbA1c (**Figure 2B**; n = 22, R² = 0.28, p = 0.0106). A similar trend was observed for fasting glucose (**Figure 2C**; n = 22, R² = 0.16, p = 0.0635), whereas no association was found with fasting insulin (**Figure 2D**; n= 22, R² = 0.04, p = 0.49). In the whole cohort, baseline CELA2A expression appeared higher in subjects with diabetes compared to the non-diabetics (2.8 ± 1.0 vs. 0.76 ± 0.46, n=12 and 11, respectively), although this did not reach statistical significance (Mann–Whitney p = 0.09; log-transformed Welch’s test p = 0.06).

Consistent with the jejunal findings, serum CELA2A concentrations measured by ELISA were significantly reduced in paired samples obtained before and after RYGB (**Figure 2E**; n = 12, p = 0.0244). Preoperative levels ranged from undetectable to 1013 pg/mL (mean ± SEM 450 ± 80 pg/mL), whereas postoperative levels ranged from undetectable to 699 pg/mL (295 ± 82 pg/mL), corresponding to an approximate 35% decrease. Ten of twelve individuals exhibited reduced circulating CELA2A concentrations following surgery. The magnitude of change did not differ between patients with and without T2D. There were no associations found between CELA2A in serum and any of the glycemic measures, neither were serum and jejunal CELA2A levels associated (not shown).

Together, these findings demonstrate postoperative divergence between gastric upregulation and jejunal and systemic downregulation of CELA2A.

### Diet-induced obesity recapitulates tissue-specific regulation in mice

**Figure 3:**
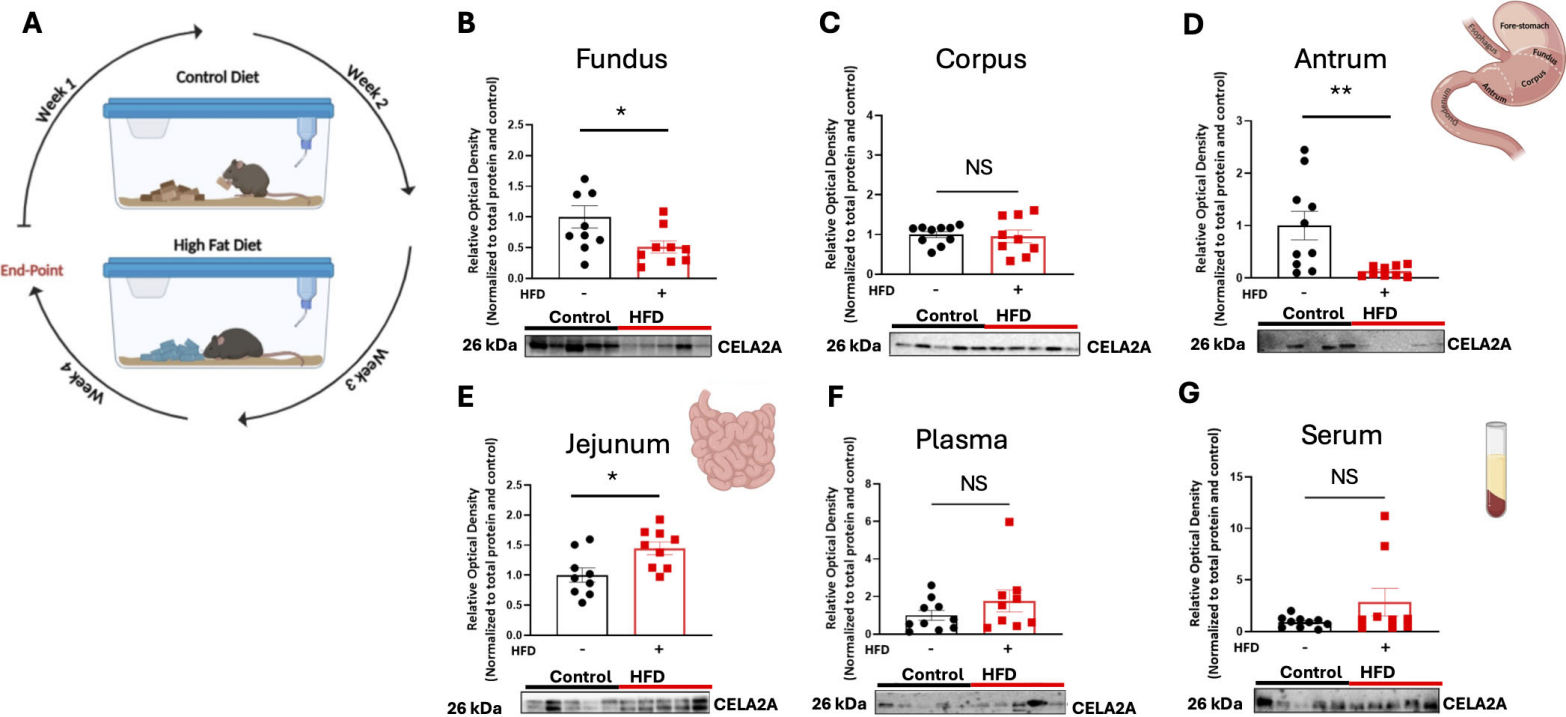
CELA2A expression in DIO mice versus control. **(A)** Experimental design: C57BL/6J mice were subject to HFD for four weeks and compared to control mice on normal chow diet. **(B)** CELA2A protein expression in fundus. **(C)** CELA2A protein expression in corpus. **(D)** CELA2A protein expression in the antrum. **(E)** CELA2A protein expression in jejunum. **(F)** CELA2A protein expression in plasma. **(G)** CELA2A protein expression in serum. Significance was calculated using unpaired t-test, n=9–10 animals/group. Mean ± SEM, *p<0.05 and **p<0.01. llustrations Created with BioRender.com.

To validate the observations obtained in obese versus postsurgical patients, we investigated CELA2A protein expression in a diet-induced obese (DIO) mouse model. C57BL/6J mice were fed either standard chow or a high-fat diet (HFD) for 4 weeks. (**Figure 3A**). Mice fed a high-fat diet (HFD) exhibited a progressive worsening of the Body Condition Score (BCS; **Supplementary Figure 2A**) and significant increase in body weight percentage over time compared to control animals on normal chow, as expected (**Supplementary Figure 2B**). The DIO mice also showed elevated blood glucose levels relative to controls, consistent with impaired glucose homeostasis (**Supplementary Figure 1C**).

To investigate the relationship between CELA2A and metabolic dysfunction, we examined its expression in the DIO and control C57BL/6J mice. CELA2A levels were analyzed in 9–10-week-old normal-weight control mice and the DIO mice (**Figure 3A**).

CELA2A expression was assessed by Western blot in gastrointestinal tissues and in plasma and serum. In the DIO mice, a band consistent with gastric CELA2A expression was significantly reduced in the fundus (p = 0.0347) and antrum (p = 0.0079), while remaining unchanged in the corpus (p = 0.7902) (**Figures 3B–D**). Conversely, jejunal CELA2A expression was significantly increased in the DIO mice (p = 0.0137) (**Figure 3E**). Even though mean circulating CELA2A levels were somewhat higher after HFD they were not significantly changed in neither plasma nor serum samples (p = 0.2293 and p = 0.1458, respectively; **Figures 3F, G**). The lack of difference observed in the blood and corpus in the mice may be attributable to the experimental conditions, as the mice had ad libitum access to food and were likely in a fed state at the time sample collection, whereas patients underwent pre-operative fasting. This difference in nutritional status may have influenced blood and corpus-specific expression levels compared to the human samples.

Thus, diet-induced obesity was associated with elevated circulating and jejunal CELA2A levels alongside reduced expression in specific gastric regions, reflecting tissue-specific regulation that partially mirrors the human findings.

### Localization of CELA2A in the upper gastrointestinal tract

**Figure 4:**
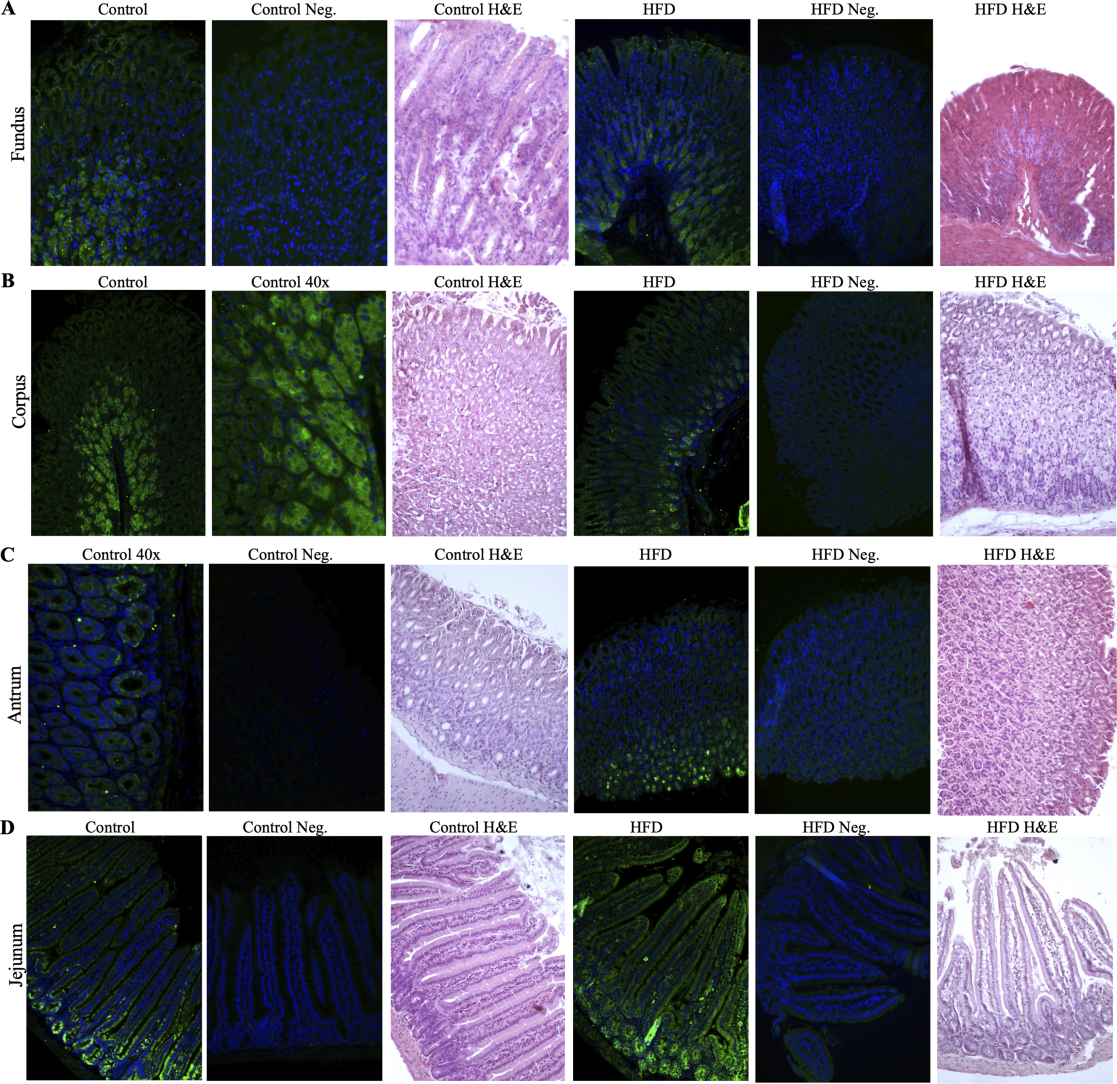
CELA2A tissue expression in the mouse stomach and jejunum. In the fundus **(A)**, corpus **(B)**, and antrum **(C)**, CELA2A expression was localized to the basal region of the gastric pits, corresponding to areas where cells display more intense hematoxylin–eosin (H&E) staining. In the jejunum **(D)**, CELA2A expression was observed within the intestinal glands (crypts) as well as in the surface enterocytes of the villi. The staining pattern was comparable between mice maintained on the control diet and those fed a high-fat diet (HFD). Magnification 20x unless otherwise stated. Negative control (Control neg): staining performed without primary antibody.

CELA2A localization was evaluated exclusively in mouse tissue, as no commercially available antibody validated for immunohistochemistry in human samples is currently available. In the mouse stomach, CELA2A expression was predominantly localized to the basal region of the mucosa within the cytoplasm of compact glandular cells. This pattern was consistently observed across the fundus, corpus, and antrum and corresponded to regions of more intense hematoxylin–eosin staining. Histological examination revealed preserved mucosal architecture, with no major structural differences between normal-weight and DIO mice (**Figures 4A–C**).

In the jejunum, CELA2A was detected both within the intestinal glands (crypts) and in surface enterocytes of the villi. Following HFD exposure, the small intestinal mucosa demonstrated marked lipid accumulation (**Figure 4D**). Negative control sections processed without primary antibody showed no detectable staining, confirming the absence of autofluorescence and non-specific background signal.

## Discussion

The present study demonstrates tissue-specific and context-dependent regulation of CELA2A in obesity and following Roux-en-Y gastric bypass (RYGB). Specifically, CELA2A expression increased in the gastric mucosa after surgery, while jejunal expression and circulating levels decreased. Jejunal CELA2A levels in untreated obese subjects were associated with HbA1c, but not with fasting insulin. HFD-induced obesity in mice was associated with increased jejunal CELA2A expression and reduced gastric expression. Together, these findings suggest that CELA2A is regulated in relation to metabolic state and nutrient exposure across different gastrointestinal compartments.

Consistent with our prior proteomic observations, gastric CELA2A expression was significantly upregulated postoperatively in the fundus and corpus, and now also confirmed in the antrum by Western blot (17). This upregulation suggests adaptive remodeling of the gastric mucosa following RYGB. The bypassed stomach is no longer exposed to luminal nutrients, which may influence local proteomic regulation. Although speculative, this interpretation is consistent with the concept that CELA2A regulation may be sensitive to nutrient exposure and gastrointestinal compartmentalization, fitting within—but not limited to—the framework of the foregut and hindgut hypotheses (11).

In contrast, jejunal CELA2A expression decreased after RYGB, particularly among patients with type 2 diabetes (T2D). Preoperative expression was primarily observed in individuals with obesity and T2D, suggesting a potential association between jejunal CELA2A expression and metabolic dysregulation. The subsequent postoperative decline may therefore reflect improved metabolic status rather than direct surgical effects per se. The stronger association between jejunal CELA2A and HbA1c, compared to f-glucose and the absence of correlation with insulin may indicate that CELA2A reflects longer-term metabolic status rather than acute insulin-dependent regulation.

Circulating CELA2A concentrations declined following RYGB. Elevated preoperative levels in obese individuals with T2D, suggest that systemic CELA2A may be associated with metabolic dysfunction. However, circulating CELA2A levels were not consistently altered across experimental models, and no direct correlation between circulating and tissue levels was observed, indicating that systemic measurements may not fully reflect local intestinal or gastric regulation.

The mouse model further supports context-dependent regulation. In HFD-fed mice with (short-term) diet-induced obesity and metabolic stress, jejunal CELA2A expression was increased, whereas gastric expression was reduced. Despite the obvious differences between this mouse model of obesity and human obesity in temporal terms, this concordance may support the interpretation that CELA2A expression is responsive to metabolic environment rather than species-specific factors. The opposing gastric and jejunal responses further highlight the compartmentalized regulation of gastrointestinal tissues in obesity and after surgical intervention.

Immunohistochemical analysis demonstrated CELA2A immunoreactivity in mucosal glandular structures in the stomach and in epithelial compartments of the jejunum. While this distribution is consistent with a potential role in mucosal or secretory processes, detailed cellular localization in human tissue was not established, and the functional implications remain to be clarified.

Taken together, these findings indicate that CELA2A is differentially regulated across gastrointestinal compartments in obesity and following RYGB. These tissue-specific patterns, together with the association between jejunal CELA2A expression and glycemic control, are consistent with a potential role for CELA2A in intestinal metabolic signaling (20). However, the present data do not establish a causal or endocrine role, and the cellular source and mechanisms of action remain to be defined.

Despite these limitations, the observed regulation of CELA2A in the proximal gastrointestinal tract is consistent with the concept of a nutrient-responsive signal potentially contributing to metabolic adaptation after bariatric surgery. In particular, the reduction of jejunal CELA2A expression after RYGB, together with its increase in diet-induced obesity, aligns with the proposed involvement of proximal intestinal factors in modulating enteroendocrine function. These findings suggest that CELA2A may represent a candidate component of gastrointestinal signaling pathways relevant to glucose homeostasis, warranting further studies to define its cellular origin and functional role.

Several limitations should be acknowledged. The postoperative gastric biopsies were obtained from a separate cohort of patients, were non-paired, and collected over variable intervals. Sample numbers were necessarily limited due to the invasive nature of postoperative gastrointestinal biopsies obtained by balloon enteroscopy in anaesthetized RYGB patients. Western blot analyses provide semiquantitative assessment of protein expression, and functional assays were not performed to directly establish mechanistic effects. Subgroup findings in patients with T2D should therefore be interpreted cautiously. Given the sequence similarity among elastase family members, antibody-based detection may not fully distinguish between CELA2A and closely related isoforms, and cross-reactivity cannot be excluded. The present study should be considered exploratory and hypothesis-generating, and warrants further mechanistic investigation.

In summary, CELA2A exhibits opposing regulation in gastric and jejunal compartments and divergent systemic patterns in obesity and following RYGB. The association with HbA1c suggests relevance for metabolic regulation. The coordinated changes observed in humans and diet-induced obese mice suggest that CELA2A responds dynamically to metabolic context. Future studies will be required to determine whether CELA2A actively contributes to metabolic adaptation or instead reflects tissue remodeling secondary to altered nutrient exposure.

## Data Availability

The proteomic analyses of the gastric mucosa from the fundus, corpus, and antrum are cavailable at PRIDE (http://www.ebi.ac.uk/pride) under the accession number (PXD065508). Other data that support the findings of this study are available from the corresponding author upon reasonable request

## Supplementary material

Supplementary Figure 1 CELA2A protein expression in the gastric mucosa (fundus, corpus and antrum) from RYGB operated subjects plotted as a function of time elapsed since surgery. Red dots represent the fundus, black dots the corpus and green dots the antrum of the secluded stomach. No correlation was observed between time after surgery and CELA2A expression levels in any of the different parts of the stomach.

Supplementary Figure 2 High-Fat Diet (HFD) Increases Body Weight Percentage Over Time and Elevates Blood Glucose Levels Compared to Control Mice. **(A)** Body Condition Score (BCS); **(B)** Body Weight Percentage Over Time; **(C)** Blood Glucose Levels in HFD vs. Control Mice. Significance was calculated using unpaired t-test, n=9–10 animals/group. Mean ± SEM, * p<0.05 and ** p<0.01.
