# Supplementary figures and images for: Opposing gastric and jejunal regulation of CELA2A in obesity and after Roux-en-Y gastric bypass suggests a role in gastrointestinal metabolic signaling
Source: Front Endocrinol (Lausanne). 2026 May 25;17:1833946. doi: 10.3389/fendo.2026.1833946 (PMC13243119; doi:10.3389/fendo.2026.1833946)

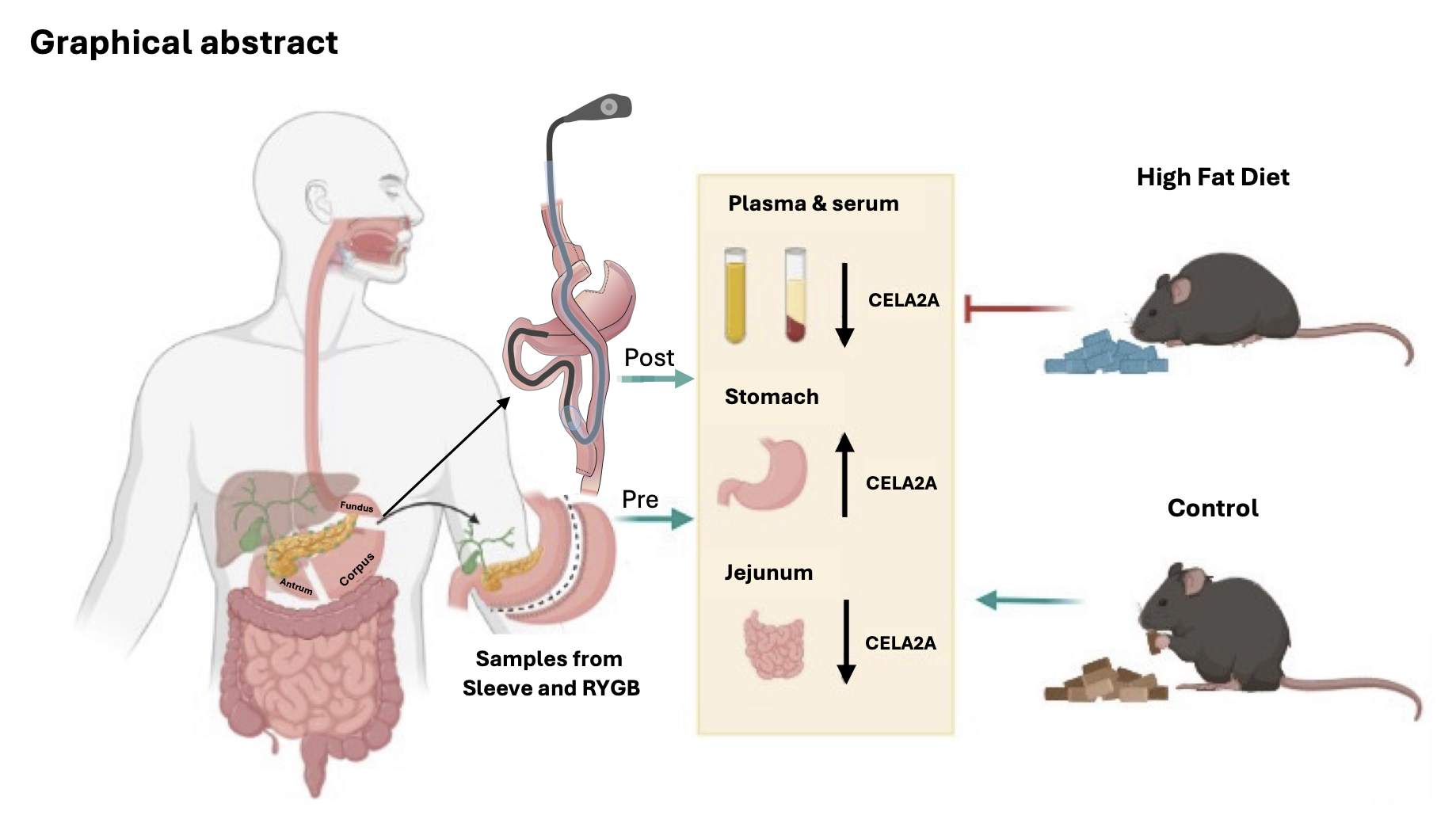

Supplement: Supplementary Figure 1 — CELA2A protein expression in the gastric mucosa (fundus, corpus and antrum) from RYGB operated subjects plotted as a function of time elapsed since surgery. Red dots represent the fundus, black dots the corpus and green dots the antrum of the secluded stomach. No correlation was observed between time after surgery and CELA2A expression levels in any of the different parts of the stomach. [file Image1.tiff]

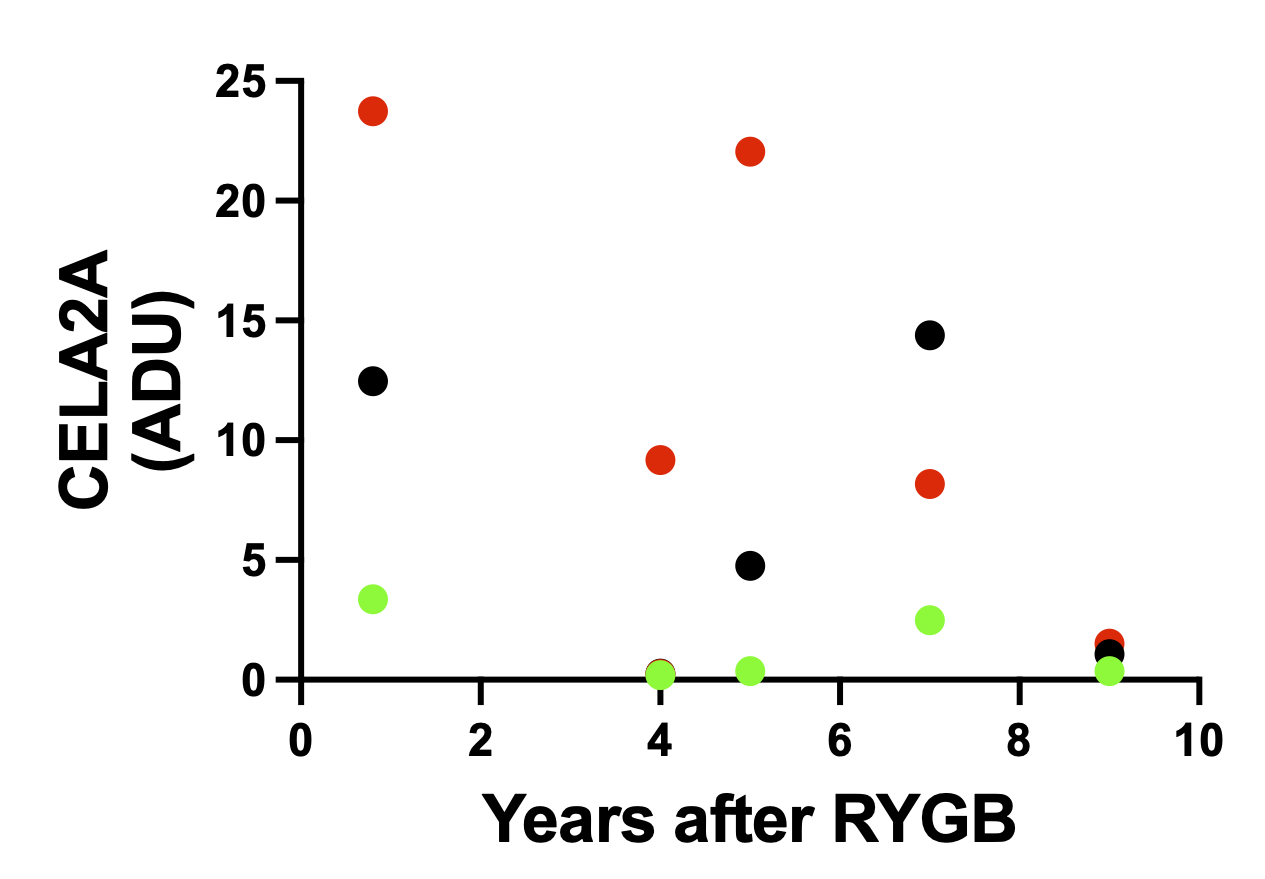

Supplement: Supplementary Figure 2 — High-Fat Diet (HFD) Increases Body Weight Percentage Over Time and Elevates Blood Glucose Levels Compared to Control Mice. (A) Body Condition Score (BCS); (B) Body Weight Percentage Over Time; (C) Blood Glucose Levels in HFD vs. Control Mice. Significance was calculated using unpaired t-test, n=9–10 animals/group. Mean ± SEM, * p<0.05 and ** p<0.01. [file Image2.tiff]

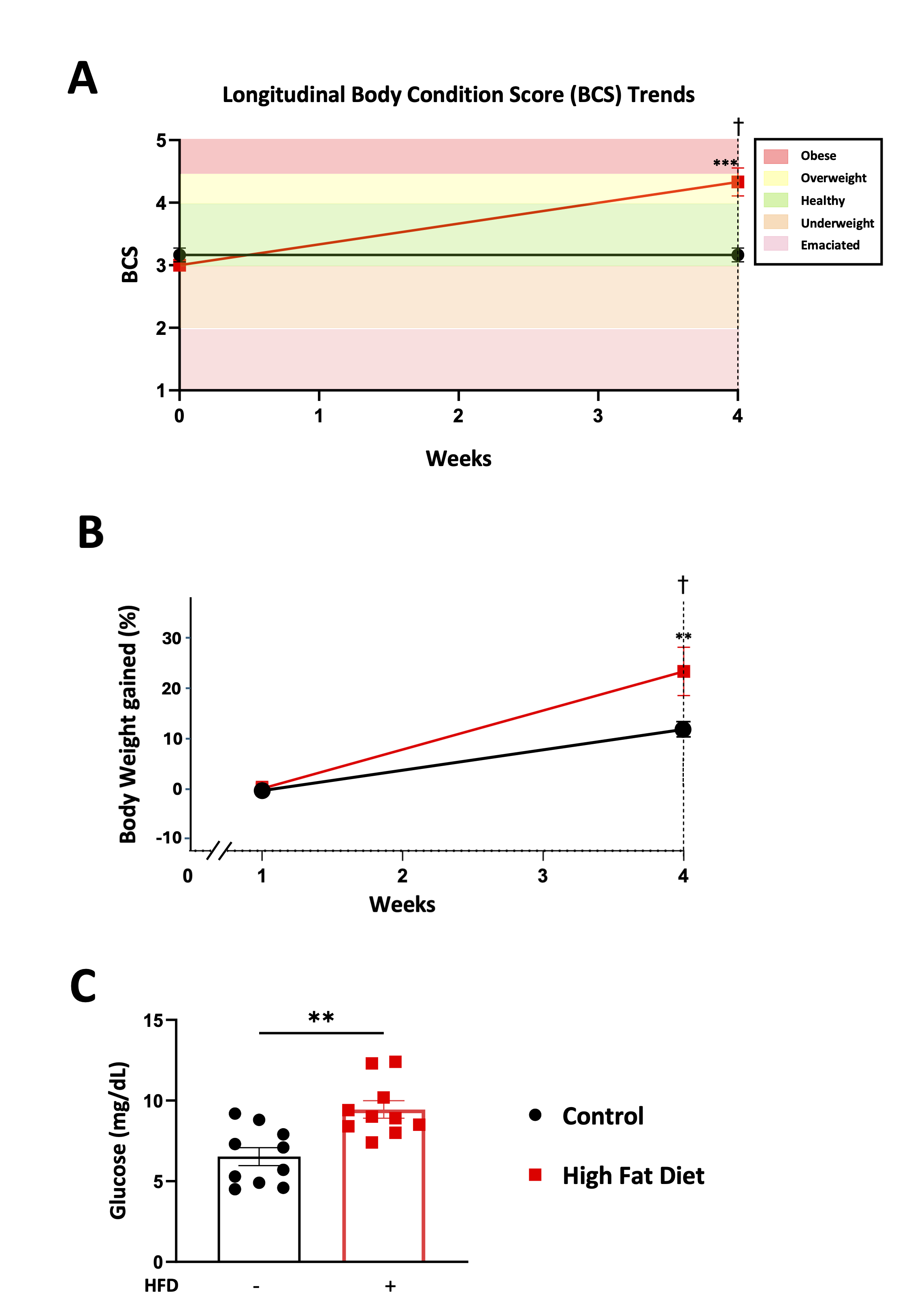

Supplement: Supplementary file 3 [file Image3.tiff]
